# Study on the stability and antioxidant effect of the *Allium ursinum* watery extract

**DOI:** 10.1186/1752-153X-7-21

**Published:** 2013-02-01

**Authors:** Salomeia Putnoky, Angela Caunii, Monica Butnariu

**Affiliations:** 1 Faculty of Medicine, Department of Hygiene, “Victor Babes” University of Medicine and Pharmacy, Bd. Victor Babes, no. 16, 300226, Timisoara, Romania; 2 Faculty of Pharmacy, “Victor Babes” University of Medicine and Pharmacy, 2A Eftimie Murgu Square, Timisoara, 300041, Romania; 3 Chemistry and Vegetal Biochemistry, Banat’s University of Agricultural Sciences and Veterinary Medicine from Timisoara, Calea Aradului no. 119, 300645, Timisoara, Romania

## Abstract

**Background:**

Organosulfur compounds usually present a reduced stability especially in the presence of oxygen. This research aimed to study the stability and antioxidant potential of the *Allium ursinum* watery extract.

**Results:**

The decrease of the antioxidant capacity verifies an exponential relation which may be formally associated to a kinetically pseudomonomolecular process. The exponential regression equation allows the half-life of the degradation process to be determined, this being 14 hours and 49 minutes in a watery environment at room temperature.

**Conclusions:**

The watery extract of *Allium ursinum* changes its proprieties in time. This might be explained by the network of hydrogen bonds in a watery environment which has a protective effect on the dissolved allicin molecule.

## Background

The species belonging to the *Allium* family have been used for a long time as a remedy for the prevention and treatment of certain diseases [1]. The adjectives associated to these plants i.e. spicy, imposing, distinct and even the latin name *Allium*, deriving from the Celtic “all” meaning pungent, reflects the presence of certain flavours and scents, all sharing a single element: sulfur. Many of these sulfur compounds contain the allyl group, name deriving from *Allium*. The very presence of these organosulfur compounds defines the character of this species. The wide spectrum of therapeutic actions has been attributed to organosulfur substances [2,3]. The chemistry of *Alliaceae* offers examples of organosulfur compounds with an amazing physiological activity, organosulfur intermediate compounds with unusual bonds [4,5], challenging analytical problems, stereochemical characteristics related to the presence of sulfur, unusual organosulfur heterocycles with important spectroscopic properties, redox reactions involving sulfurs, pericyclic reactions in organo-synthetical chemistry [6,7]. The first exhaustive synthesis on these compounds was published in 1992 [8]. This paper, besides presenting an impressive number of chemically characterised compounds, confirms previous data according to which the main component with a therapeutic action is allicin [9,10]. *A. ursinum* (ramson) and other representatives of the *Allium* species, contain 1–5% nonprotein secondary metabolites of aminosulfuric acids [11]. In the cell, the stable precursor of the antibacterial principle of Cavallito, the S–oxyde (+/−) of S–2–propenyl cysteine and the S–oxyde of S–alkenyl cysteine (odour and flavour precursors) are located in the cytoplasm while the enzyme alliinase in the vacuole [12]. Both in the paper by Block [13] as well as in previous published works, two beneficial properties of this component are highlighted: the antibacterial effect and the antioxidant capacity (the property to bind reactive free radicals) [14,15]. Using chromatographic assays, the active components were isolated and subsequently identified. Analyses by high-performance liquid chromatography suggested that these compounds were sulfur components, with a characteristic absorbance at 250 nm. Gas chromatography–mass spectrometry analyses allowed the chemical structures of the isolated components to be investigated [3,16]. Ramson, garlic and onion extracts have been used in popular medicine, and commercial products of these plants record an increasing use.

According to epidemiologic evidence, low cancer risks are associated to a high intake of alliaceae [17,18], reason for which health organizations recommend the use of *A. ursinum* as raw material in diets aiming to prevent cellular malignancy. For all these reasons, a detailed research of the organosulfur chemistry of the genus *Allium* seems justified [19,20]. Literature data states that in a watery solution, allicin presents an increased stability as compared to other solvents (e.g. alcohol, acetone etc.) [21].

The purpose of the present study is the watery extract of *A. ursinum*. This research intends to study the stability of the allicin containing watery extract of *A. ursinum*.

The degradation in time of this compound affects the stability and the period of use of ramson preparations, and as such, a significant study of this degradation process is not without interest.

## Results

### The spectrophotometric study of the extract

**Figure 1:**
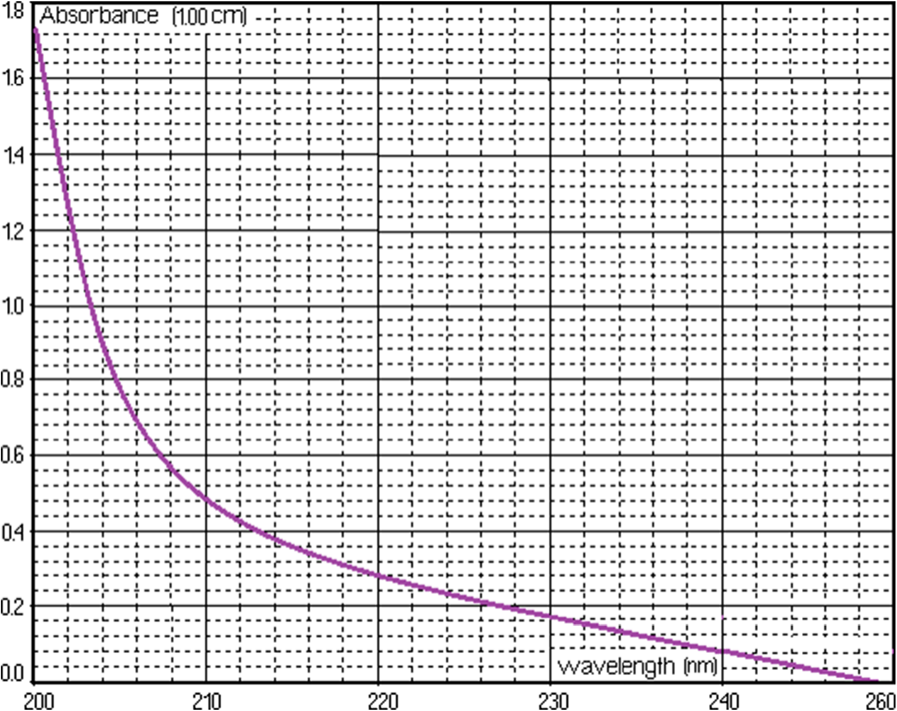
**The UV absorption spectrum of the *A. ursinum *solution.**

The UV absorption spectrum of the *A. ursinum* solution, obtained against distilled water in a 1 cm quartz cuvette, is presented in Figure 1. The profile of the absorption spectrum is significant in assessing the areas of highest and lowest absorption, and distinct profiles can signify the effects of different molecules. Using the literature value of the specific absorption at 240 nm (145.4 dL/g · cm), the allicin content reported to fresh raw material was determined: 0.694 μg/ml.

### Determination and characterization of the antioxidant capacity

**Figure 2:**
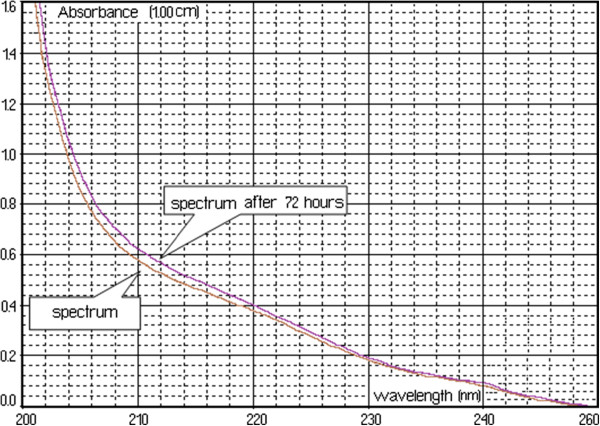
**Spectrum change of the watery extract of *A. ursinum *in time.**

**Figure 3:**
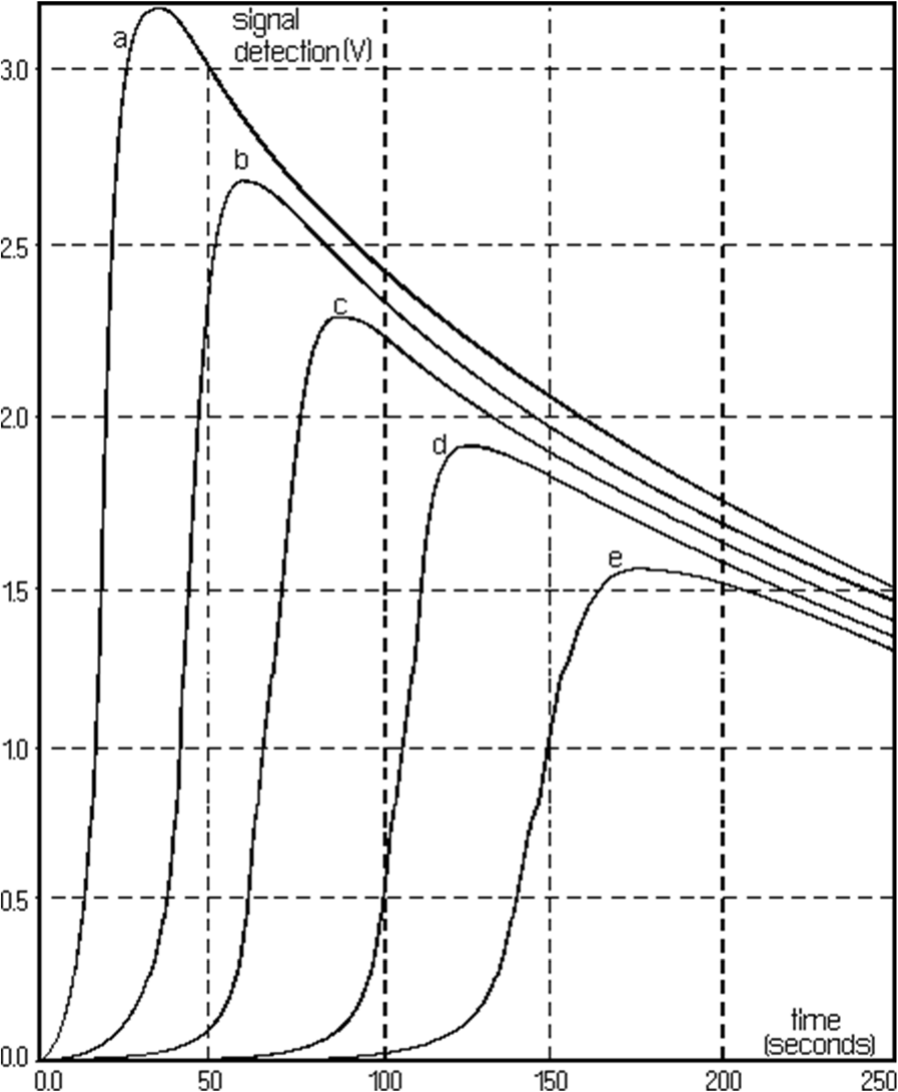
Curves a–e corresponding to increasing antioxidant quantities.

**Figure 4:**
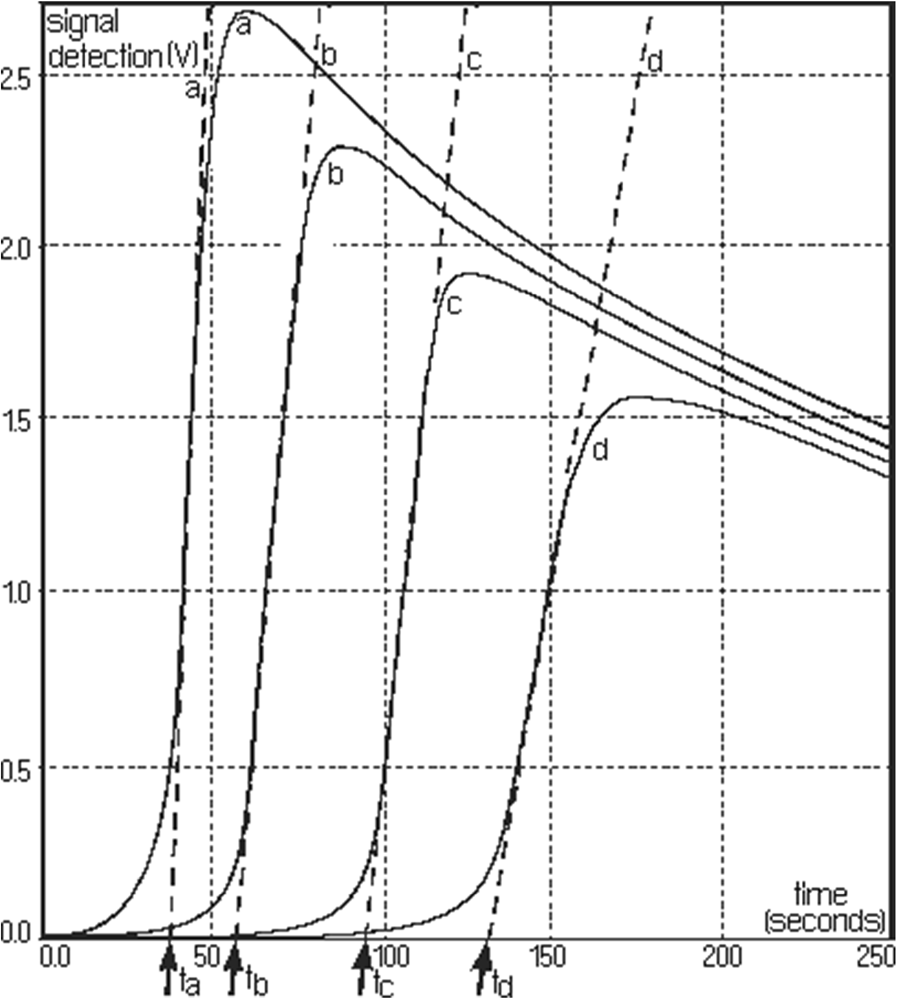
The algorithm for determining induction times.

Figure 2 shows spectrum change of the watery extract of *A. ursinum* in time, with the generation sequence of reactive oxygen species (singlet oxygen and superoxide anion radical). The evolution of the emitted light intensity (expressed as detector signal) is recorded on a curve shaped as shown in Figure 3. The curves a–e in Figure 3 corresponds to increasing quantities of antioxidant in the system. It may be observed that, with the increase in the amount of antioxidant, the ascendant branch of the curves is preceded by increasing induction intervals. Figure 4 presents a group of curves (a–d) obtained with the help of a series of standard solutions. The values of induction times (IT) and effective induction times (EIT) as well as the conventional expression of the antioxidant character (vitamin C nmol equivalents) are presented in Table 1. The antioxidant character of the analysed products is obtained by comparing the curves associated to these samples with those obtained with the help of standard solutions. Following the described studies, it has been observed that the watery extract of *A. ursinum* changes its properties in time. Despite the fact that this change, is not very visible in the UV absorption spectrum (Figure 1), it becomes evident in the study on the antioxidant capacity. The induction time corresponds to the intersection between the time axis and the tangent to the respective curve, at its inflection point.

**Table 1:** Values of induction times (IT) and effective induction times (EIT)

**TI (s)**	**TIE (s)**	**nmol vitamin C**
22.9	0.0	0.0
55.8	32.9	0.5
85.4	62.5	1.0
112.1	89.2	1.5
136.3	113.4	2.0

**Figure 5:**
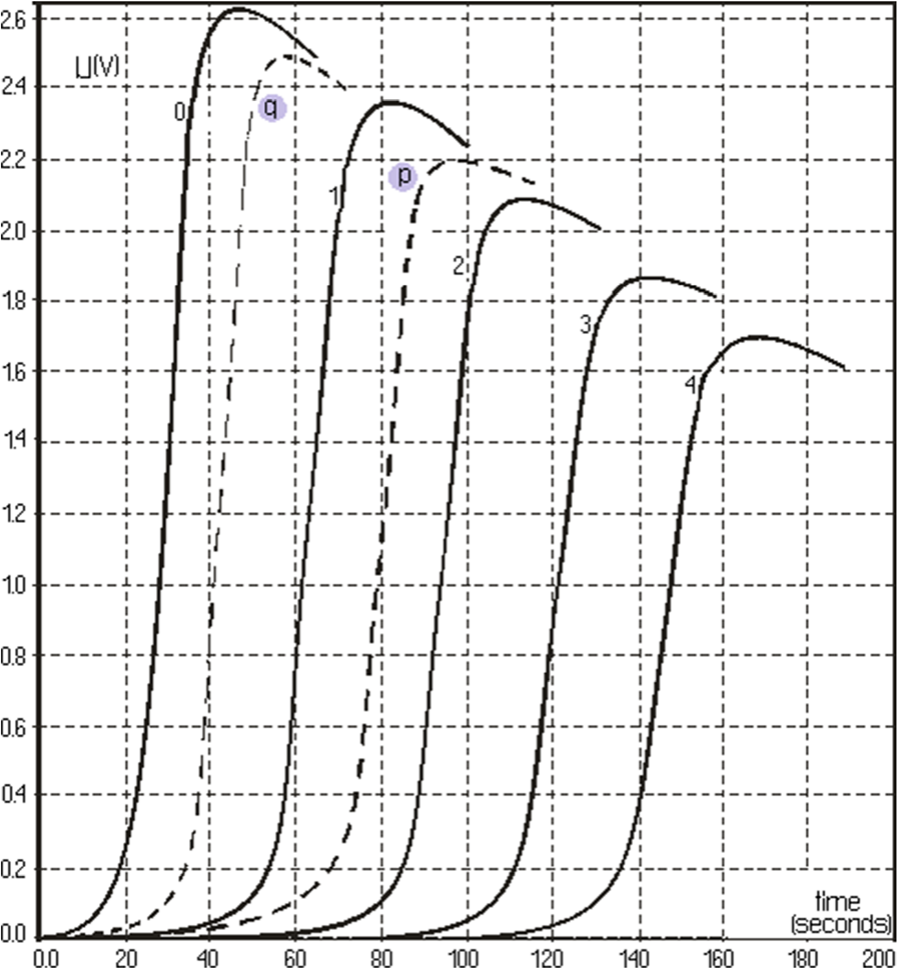
**Curves p and q corresponding to fresh and 24 h at room temperature *A. ursinum *extracts, respectively.**

**Figure 6:**
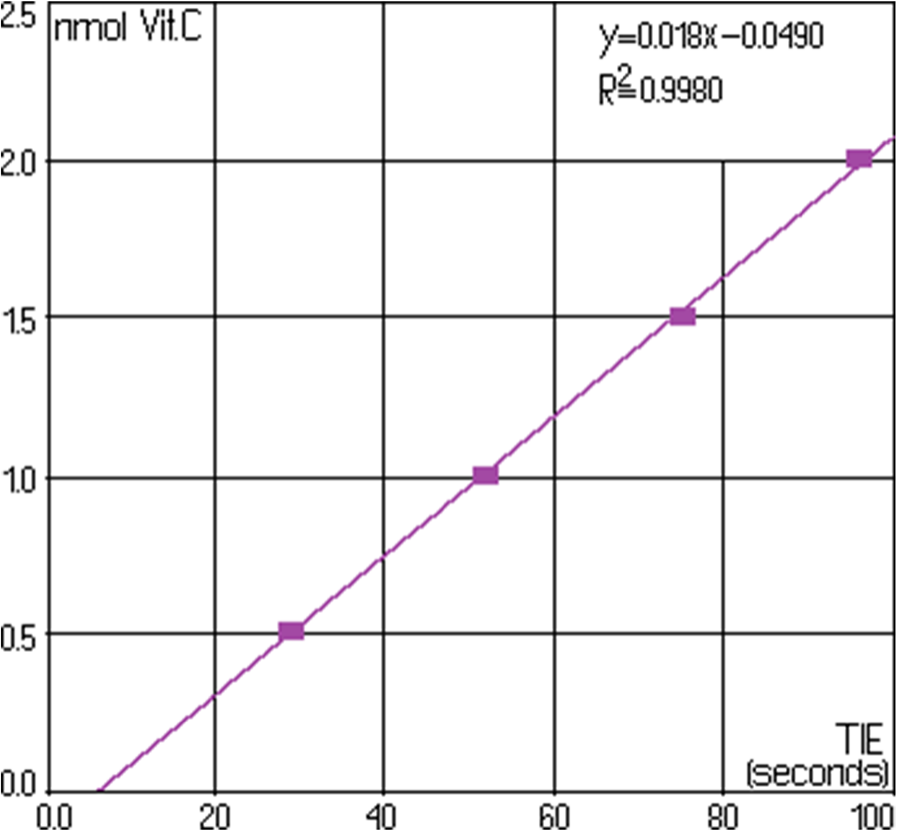
Data on the series of standard solutions (linear relationship).

The curves p and q in Figure 5 correspond to fresh and 24 hours at room temperature *A. ursinum* extracts, respectively. They correspond to the first and last lines in Table 2, respectively. Intermediate samples, included in Table 2, generate luminiscence curves between those labelled with p and q in Figure 5. Data on the series of standard solutions, included in Table 1, acceptably confirm a linear relationship (Figure 6).

**Table 2:** **Decrease in time of the antioxidant activity of the *A. ursinum *watery extract**

**Time of degradation (ore)**	**TIE (s)**	**Nmol vitamin C**
0	45.4	0.75
3	39.2	0.64
6	34.0	0.55
9	29.1	0.46
12	25.6	0.40
15	22.5	0.35
18	19.7	0.30
21	17.0	0.25
24	14.5	0.21

**Figure 7:**
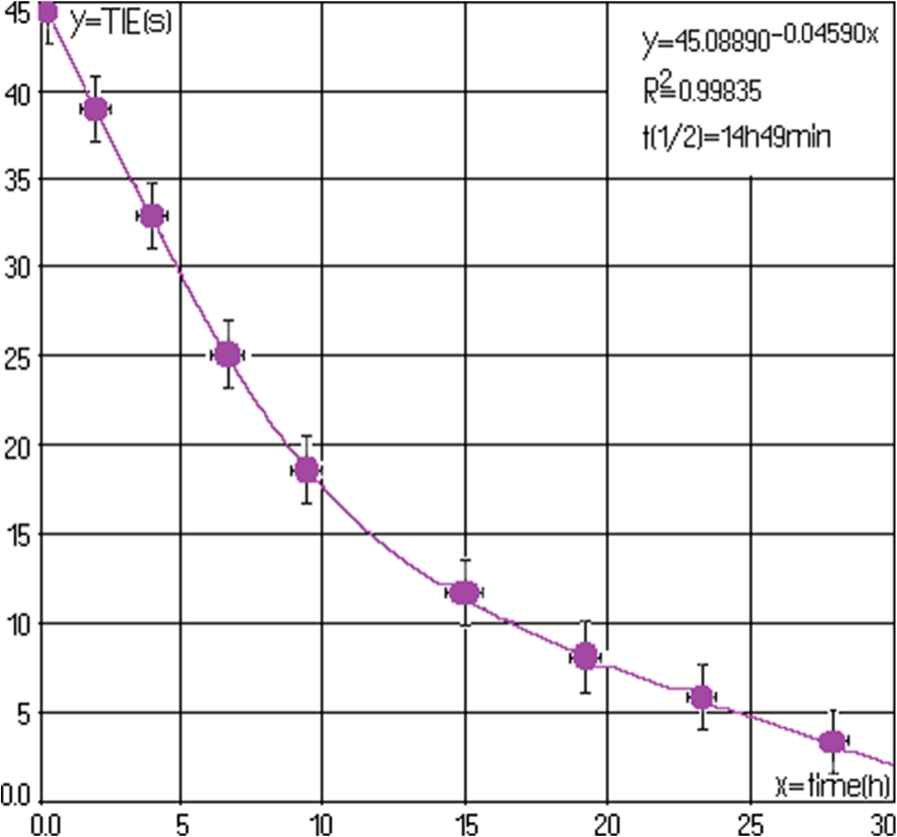
**Decrease in time of the antioxidant activity of the watery extract of *A. ursinum.***

Table 2 and Figure 7 present in figures and graphs, respectively, the decrease in time of the antioxidant activity. The decrease in the antioxidant activity confirms an exponential relation which formally may be associated to a kinetically pseudo-monomolecular process. The exponential regression equation allows the half-time of the degradation process to be determined, this being of 14 hours and 49 minutes in a watery environment at room temperature.

## Discussion

Using the literature value of the specific absorbance at 240 nm (145.4 dL/g · cm), the allicin content reported to the used raw material could be determined i.e. 0.694 μg/ml.

**Scheme 1:**
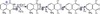
**Process [A]–obtaining the singlet state (1 s) of the molecule; process [B]–process the molecule goes into the triplet; process [C]–generating the singlet oxygen reactive species; process [D]–the generation of an anion radical in doublet state (**^**2**^**S).**

**Scheme 2:**
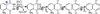
Process [E], [F] and [G]– the formation of the superoxide anion; processes [H]– transforms into the acid dianion.

The determination of the antioxidant capacity is based upon photochemical generation initiated by an UV radiation in the 185–240 nm domains, of the anion (O^–^ _2_**◦**) superoxide radical. The sequence presented in Schemes 1 and 2 includes the optical excitation of the free radical generator (damnacantal) thus obtaining the singlet state (1 s) of the molecule (process [A]). By an “inter system crossing”-type process (process [B]), the molecule goes into the triplet state which, due to known molecular spectroscopy selection rules, is stable enough to react either with oxygen in the normal triplet state (generating the singlet oxygen reactive species) (process [C]), or with a reducing (also called a reductant or reducer) agent (mono-electronic reduction process) (process [D]) with the generation of an anion radical in doublet state (^2^S) and the formation of the superoxide anion (process [E]). The latter, by a series of processes ([A]–[H]), transforms into the amino orthophthalic acid dianion, in the singlet state, which, by reversal process to the fundamental state, emits a light beam in the 425–450 nm spectral domain [22]. Antioxidants from the captured samples yield superoxide ions and reduce the radiation intensity generated by excited luminol (luminol reaction inhibiting-blank). Standard soluble compounds are TROLOX (derivate of α–tocopherol), and for the water–soluble, ascorbic acid.

For both it performs a calibration curve and estimate the advance between the integral under the curve of the blank (solution without antioxidant) and sample (standard solution or extract with antioxidant-plant) and distribute by the standard integral.

These estimates are done automatically by the software unit.

The intensity of the light signal, measured by a photomultiplier, depends on the speed of the processes [F] and [G]. If the system does not include a compound capable to bond free radicals, the entire amount of generated anion superoxide is consumed by the light supplying agent “luminol”, and the intensity of the emitted light is maximal.

If the system contains an amount of free radical binding agents (antioxinants) a competition between luminol and the free radical binding agents occurs for the superoxide anion radical. In this case the light signal detected by the photomultiplier has a lower intensity [23]. The unimolecular decomposition of methyl methane thiosulfate is favoured by the weak S–S bond which has the energy of 46 +/−4.6 kcal/mol comparable to the energy for the dissociation of the S–S bond in dimethyl trisulfure but considerably lower than in dimethyl disulfide (75 kcal/mol) [24].

By comparison, the S–S bonding energies in phenyl benzene tiosulfinate and diphenyl disulfur are 35 and 65 kcal/mol, respectively. The oxygen in tiosulfinate participates in the hydrogen bond, even if not as strong as the oxygen in sulfoxydes [25]. By using the strength of the hydrogen bond, metan tiosulfinate, ethylethane thiosulfinate and dimetyl sulfoxide were demonstrated to cause 278, 308 and 360 cm^–1^ changes, respectively, in the vibration frequency of the bond. In order to answer the question on the way to benefit from an *Alliaceae* diet, the following recommendations may be formulated: The texture of *Alliaceae* species is completely crushed when these are consumed, thus allowing a large amount of S–oxyde S–alkenylcysteine to enter unchanged into the intestinal tract. As alliinase are ireversibly inactivated by the gastric pH, sulfoxides are cleaved by intestinal bacteria into corresponding disulfures [26]. Thiosulfinates which reach the stomach survive the low pH long enough to attack existing pathogenic microorganisms. The allk(en)yltiolation of certain SH biological groups by thiosulfinates, di– or polysulfures or ajoene is beneficial (tiamine allyl tiolation occurs rapidly with the generation of allyl tiamine which is easier absorbed from the intestine than tiamine). The disulfures and other sulfur compounds formed in *Alliaceae* species play a role in the intestinal tract by deactivation of nitrites or other environmental toxins [27]. The metabolic pathway of *Alliaceae* organosulfur compounds represents an important field for future research.

## Conclusions

The study confirms the literature statement that the *A. ursinum* watery extract has an antioxidant capacity lasting for several hours. The half-time, as experimentally determined in this study, is 15 hours and 45 minutes at room temperature.

As such, after 24 hours, the antioxidant capacity of an *A. ursinum* watery extract is decreased. Observing the degradation process in time, an exponential relation was described which may formally correspond to a kinetically pseudomonomolecular model. Even if absorbance reported to specific wavelengths correspond to the domain indicated in literature for the allicin component, the lack of a well-defined chromophore in the allicin molecule does not allow an effective monitoring of the degradation process. Unlike the UV study, monitoring the antioxidant capacity by chemiluminescence method represents a rapid, comfortable and adequate technique for monitoring allicin changes in a watery environment.

## Methods

### Equipments and substances

UV absorption spectra were recorded with a PG Instruments UV–VIS spectrophotometer using the UV WIN 5.05 software. The antioxidant capacity was determined by chemiluminiscence using the “PHOTOCHEM” dedicated device produced by Jena Analytic, Germany. The standard substances were purchased from Sigma Aldrich.

### Obtaining the extract

A fresh ramson bulb was collected from April to May in deciduous forests (western part of Romania) weighed with the semi micro analytical scale (7.00 g) is crushed in a grinding mortar and homogenized in 50 mL distilled water. The homogenized matter is quantitatively transformed in a 250 mL measuring bottle and the volume is completed with distilled water. The non-homogeneous content of the bottle is filtered during several stages, using filtration membranes with increasingly lower porosity (osmonics–type membranes, manufactured by Micron Separations Inc.).

### The spectrophotometric study of the extract

The filtrate is diluted 30 times with distilled water to obtain the working solution. The UV absorption spectrum of the working solution was obtained against distilled water in a 1 cm quartz cuvette. In order to argument the identity of the absorbing species (allicin), the absorbance report measured at 240–254 nm is determined according to literature specifications. For the fresh watery extract, this report is 1.442, value situated in the 1.4–1.5 interval also indicated by literature data [25].

### Determination and characterization of the antioxidative capacity

Eight 2 mL aliquots from the working solution are frozen at −10°C. By successively defreezing these aliquots, the antioxidant capacity is measured every 3 hours. The determination of antioxidant capacity is based upon the photochemical generation of the superoxide anion radical initiated by a 185–240 nm UV beam.

## Competing interests

The authors declare that they have no competing interests.

## Authors’ contributions

MB conceived the study, participated in the design and co-ordination of the experiments and data interpretation and helped draft the manuscript. AC and SP produced samples, performed data analysis and data interpretation. All authors have equal rights and all authors read and approved the final manuscript.
